# Walnut ointment promotes full-thickness burning wound healing: role of linoleic acid

**DOI:** 10.1590/acb370902

**Published:** 2022-11-28

**Authors:** Dan Zhao, Jinli Xiao, Lijuan Qiang, Xingwang Deng, Jingjing An, Qing Zhang, Fang Zhao, Jiaxiang Ma, Chao Fang, Guangyu Guan, Yinsheng Wu, Yan Xie

**Affiliations:** 1 Research Assistant. Ningxia Medical University General Hospital –Tissue and Organ Bank – Ningxia, China.; 2 Graduate student. Ningxia Medical University – School of Clinical Medicine – Ningxia, China.; 3 Surgeon-in-charge. People’s Hospital of Ningxia Hui Autonomous Region – Department of Burns and Plastic Surgery – Ningxia, China.; 4 Associate Professor of Surgery. The First People’s Hospital of Shizuishan – Department of Burns and Plastic Surgery – Ningxia, China.; 5 Technologist-in-charge. Ningxia Center for Diseases Prevention and Control – Department of Physical and Chemical Examination – Ningxia, China.; 6 Technologist. Ningxia Medical University General Hospital – Tissue and Organ Bank – Ningxia, China.; 7 Surgeon-in-charge. Ningxia Medical University General Hospital – Department of Burns and Plastic Surgery – Ningxia, China.; 8 Senior Technologist. Ningxia Center for Diseases Prevention and Control – Department of Physical and Chemical Examination – Ningxia, China; 9 Professor of Surgery. Ningxia Medical University General Hospital – Department of Burns and Plastic Surgery – Ningxia, China.; 10 Professor. Ningxia Center for Diseases Prevention and Control –Tissue and Organ Bank – Ningxia, China.; 11 PhD. Queensland University of Technology – Faculty of Health – Brisbane, Australia.

**Keywords:** Burns, Skin Wound Healing, Walnut Ointment, Linoleic Acid, Keratinocytes, Fibroblasts

## Abstract

**Purpose:**

To investigate the active ingredients of walnut ointment (WO) and its mechanism in repairing wounds.

**Methods:**

The ingredients of WO were detected by gas chromatography–mass spectrometry. The effect of linoleic acid (LA) was tested by in vitro Alamar Blue (AB) reagent. Image J software, histological and immunohistochemical analysis were used to confirm the healing effect of LA in the porcine skin model. The animals were euthanized after the experiment by injection of pentobarbital sodium.

**Results:**

LA, 24% in WO, promotes keratinocytes and fibroblasts proliferation, which were 50.09% and 15.07% respectively higher than control (p < 0.05). The healing rate of the LA group (96.02% ± 2%, 98.58% ± 0.78%) was higher than the saline group (82.11% ± 3.37%, 88.72% ± 1.73%) at week 3 and week 4 (p < 0.05). The epidermal thickness of the LA was 0.16 ± 0.04 mm greater and the expression of the P63 and CK10 proteins was stronger in the LA group than the control (p < 0.05).

**Conclusions:**

LA, which is the main components in WO can promote full-thickness burning wounds (FBWs) by stimulating cell proliferation and differentiation.

## Introduction

Burns generally refer to damage to body tissues caused by heat or certain nonthermal factors. According to the World Health Organization, about 11 million people suffer from burns every year^1^. Severe burns can lead to full-thickness wounds even nonhealing wounds due to full-thickness skin defects, long-term inflammation, and bacterial invasion, among other reasons^2^. The current clinical methods for the treatment of full-thickness burning wounds (FBWs) mainly include antibiotic treatment, negative pressure sealing drainage technology, and epidermal growth factor (EGF) treatment, among others. Long-term use of antibiotics can lead to drug resistance, which may cause allergic reactions depending on the individual. The cost of negative pressure sealing drainage technology is relatively high. EGF have become a routine clinical treatment method for wounds, but they are readily affected by the external environment and cannot guarantee the expected therapeutic effect. In summary, low-cost, stable curative treatment methods for burning wounds need to be further explored.

Knowledge of traditional Chinese medicine has been handed down for generations. After thousands of years of investigation and practice, the application of traditional Chinese medicine to burning wounds has a unique effect. Walnut is a botanical species native to China and Kyrgyzstan^2^. Walnut has antioxidant, anti-inflammatory and other effects, and can be used to treat chronic inflammatory diseases, diabetes and other diseases^3^^–^ 5.

Previous research showed that walnut ointment (WO) can significantly promote the healing of burning wounds during clinical treatment^2^. However, WO is a mixture, and its active ingredients and mechanism have not been determined. This study investigated the active ingredients of WO and elucidated its mechanism in repairing FBW, providing new theoretical support and ideas for clinical wound repair treatment programs.

## Methods

### Ethics

Ethical approval has been obtained from the ethics committee of the General Hospital of Ningxia Medical University (GHNMU) (Yinchuan, Ningxia Hui Autonomous Region, China) (approval No.: 2017-204, 2018-358). The research on animals strictly followed the ethical review of experimental animals by Ningxia Medical University (NMU).

### Component analysis

A total of 4.1642 g WO sample was weighted and extracted using petroleum ether (Sinopharm Chemical Reagent, Tianjin, China). Following the extraction, the residue was 2.4819 g crude fat. Of this crude fat, 0.1155 g was weighed and added to methanol (Merk, Darmstadt, Germany), sodium hydroxide (Sinopharm Chemical Reagent) and internal standard; then refluxed until the oil droplets disappeared. Subsequently, 8 mL boron fluoride methanol solution (15%) with trifluoride (Anpel, Shanghai, China) was added and refluxing continued for 2 min. The heating was halted. N-hexane (Merk), saturated sodium chloride (Sinopharm Chemical Reagent), and anhydrous sodium sulfate (Guangfu Chemical, Tianjin, China) were added. A Shimadzu 2010plus Gas Chromatograph (Shimadzu, Kyoto, Japan), with a DB-FFAP column (Agilent Technology, California, USA) and an Agilent 7890B-5977A Gas Chromatography Mass Spectrometer (Agilent Technology, Wisconsin, USA) with a DB-WAXms column (Agilent Technology, California, USA) were used for analysis^6^.

### Cell studies

#### The effect of linoleic acid (LA) on the proliferation of human keratinocytes (HK)

HK (China Center for Type Culture Collection, Wuhan, China) were revived and 4 × 10^3^ cells were plated in a 96-well plate. After 12 h of incubation, two different LA (Aladdin, Shanghai, China) concentrations (180 mg/mL and 112 mg/mL) were added dropwise to the 96-well culture plate. For the negative control, serum-free media (SFM) was added, while for the positive control, 10% fetal bovine serum (FBS), (Sigma, New Zealand, Australia) was added. After incubating for 48 h, images were obtained under the microscope to observe the changes in cell morphology. Alamar Blue (AB) reagent was added and the optical density (OD) value was used to represent the effects on HK proliferation in the different groups^7^.

#### The effect of LA on the proliferation of human fibroblasts (HF)

Skin tissue used in the study consisted of the residual skin from skin transplantation of burn and cosmetic surgery patients at the GHNMU. Informed consent was obtained from all patients. Following aseptic treatment, the skin tissue was mashed and the trypsin (Thermo Fisher Scientific, New York, USA) was added to the mashed tissue. Type I collagenase solution (Gibco) and the digested tissue were added to a centrifuge tube. Subsequently, the tissue mass was centrifuged and cultured in a carbon dioxide incubator. The fourth to tenth passage cells were used for this experiment^8^. Following cell counting, 3 × 10^3^ cells were plated in a 96-well plate.

Following 12 h of incubation, LA at two different concentrations (180 and 112 mg/mL) was added dropwise to the 96-well culture plate. SFM was added for the negative control while 10% FBS was added for the positive control. After incubating for 48 h, images were obtained under the microscope to observe the changes in cell morphology. AB reagent was added and the OD value was determined to investigate the effect of different LA concentrations on HF proliferation^7^.

### Animal studies

Three female Guangxi Bama minipigs aged 3 months and weighing 18–20 kg (Tianjin Bainong Experimental Animal Breeding Technology, Tianjin, China) were used. Animals were housed in isolation cages and maintained at the general laboratory for animal breeding of the Laboratory Animal Center of NMU.

#### Creation of a porcine FBW model

Full-thickness burns, also known as third-degree burns extend through both the epidermis and dermis and into the subcutaneous fat or deeper^9^. The creation of the full-thickness burn model refers to the porcine FBW model, which has been previously published^2^^,^ 10. Animals were anesthetized with xylazine hydrochloride (i.m., 0.1 mL/kg, Shengda Animal Drugs Co. Ltd., Dunhua, China), midazolam (i.v., 0.1 mg/kg, Jiangsu Nhwa Pharmaceutical Co., Ltd., Xuzhou, China) and fentanyl (i.v., 0.0025 mg/kg, Yichang Humanwell Pharmaceutical Co., Ltd., Yichang, China) after a night of fasting^2^^,^ 10. The wound area was flushed with sterile normal saline (NS) to ensure optimal thermal coupling. Nine wounds were created on the back and sides of each pig using a 265 g aluminum rod (a cylinder with a diameter of 5 cm, with a net weight of 265 g, and a 0.8 cm thick polystyrene plate is wrapped around the aluminum block to keep the temperature constant) preheated in hot water at 95 ± 0.2 °C for 10 min^10^. The heated bar was applied vertically to the skin for 35 s under a pressure of 1 kg^10^. After surgery, bandage and fix with cotton pad accessories. The animal was treated with cefoperazone (i.v., 50 mg/kg, Zhongnuo Pharmaceutical Co., Ltd., Shijiazhuang, China) to prevent infection, and awoke with Analeptics (i.m., 0.1 mL/kg, Shengda animal drugs Co., Ltd., Dunhua, China). Food and oral rehydration salts could be given 3 h after awakening^2^^,^ 10. The wounds were incised 4 days later and treated with medicine 1 week later.

#### Treatments

Each piglet had nine wounds divided into three groups. In the LA group, approximately 200 μL LA was applied to the wound surface. In the EGF (Haohai Biological Technology, Shanghai, China) group, EGF was applied to the wound surface by soaking a double layer of dry gauze with EGF at a concentration of 5000 IU/mL/10 cm^2^. The NS group was covered with gauze soaked in 0.9% sodium chloride solution. Treatments were applied every 2 days until the wound healed.

#### Wound healing valuation (overall)

The images of the wounds were obtained using a self-made isometric photographing aid every week up to 4 weeks after medication. When the wound surface and the surrounding skin were cleaned with NS, the area was measured from the images using Image J software (NIH, USA)^10^.

#### Healing time and survival analysis of FBW

Wound healing time was the time taken to reach complete epithelialization. Twelve wounds of animals were treated with LA, EGF and NS respectively and recorded for statistical analysis. Survival rate was estimated by the Kaplan–Meier method^11^. Wound existence was defined as *survival* and 100% healed of wound as *death*.

#### Wound healing valuation (histological and immunohistochemical staining)

To determine the thickness of the epidermis after the wound was healed, five fields of view were randomly selected to obtain images under a microscope (10×), and measured with Image J (NIH, USA) software to analyze the epidermal thickness. Samples were taken after wound healing, and immunohistochemical staining was used to show the expression of protein P63 (Biocare Medical, CA, USA) and Cytokeratin10 (CK10, Abcam, Cambridge, United Kingdom). Image-Pro Plus 6.0 software (Media Cybernetics, USA) was used to determine the OD of the positive expression of the proteins^10^.

### End points and follow-up

After the cell experiment, the cells were treated with sodium hypochlorite solution (10%). The animals were euthanized immediately after the experiment by intravenous injection of pentobarbitone sodium (100 mg/kg, Sihuan Pharmaceutical Factory, Beijing, China) and handed over to the Laboratory Animal Center of NMU for centralized processing^10^.

### Statistical analysis

All data were analyzed with SPSS 18.0 (SPSS Inc., Chicago, IL, USA) software. One-way analysis of variance and Tukey’s post-hoc test were used to determine statistically significant differences between treatment groups. P < 0.05 was considered statistically significant^2^.

## Results

### Determination of LA content

**Figure 1:**
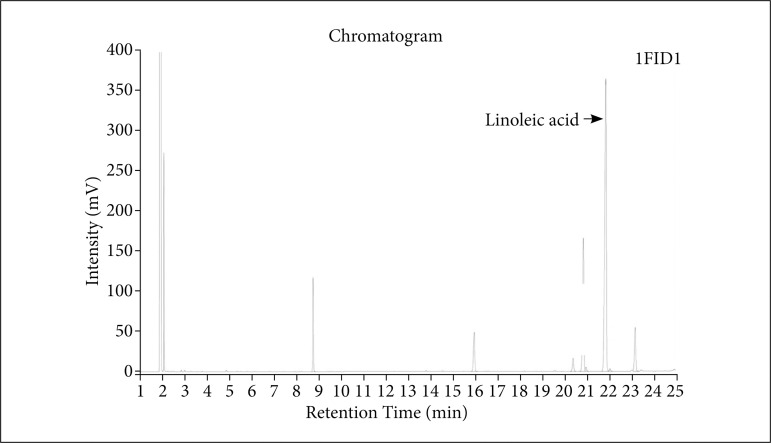
Quantitative and qualitative analyses spectrum of the WO. The compound indicatedby the arrow is LA. Other irrelevant signals have been removed from the image.

An internal standard method showed that the percentage of total fatty acids was 73% ± 4%, of which the LA content was 40.1% ± 0.8%, such that the LA content in the ointment was approximately 24% (Fig. 1, Table 1).

**Table 1:** Peak table.

Compound	Retention time (min)	Area (mV×min)	Height (mV)	Concentration (mg/mL)
LA	21.814	1,762,387	363,884	5.334

LA: linoleic acid.

### Cell studies

#### The effect of LA on HK proliferation

**Figure 2:**
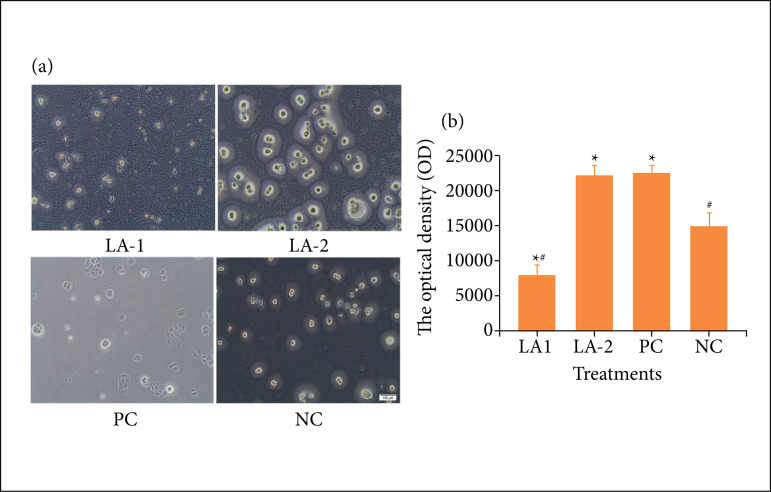
The effect of LA on HK proliferation. **(a)** The proliferation of HK underthe microscope; **(b)** The effect of LA on the HK proliferation rate (n = 3).

LA-1: LA at a concentration of 180 mg/mL; LA-2: LA at a concentration of 112 mg/mL; PC: Positive control (serum-containing medium); NC: Negative control (SFM). Significance of the differences between the NC and HK were set at * p < 0.05 or ** p < 0.01. Significance of the differences between the PC and HK were set at ^#^ p < 0.05 or ^##^ p < 0.01.

When different concentrations of LA were applied to HK, cell density was different compared with the negative and positive controls (Fig. 2a). The cells in the positive control group were dense and aggregated. In the experimental group with an LA concentration of 112 mg/mL, the number of cells was increased compared with the negative control group. By contrast, in the experimental group with an LA concentration of 180 mg/mL, the number of cells was relatively reduced than the experimental group with an LA concentration of 112mg/mL (Fig. 2a). The results of the AB test showed that an LA concentration of 112 mg/mL promoted cell proliferation, which were 50.09% higher than the negative control group (n = 3, Fig. 2b).

#### The effect of LA on HF proliferation

**Figure 3:**
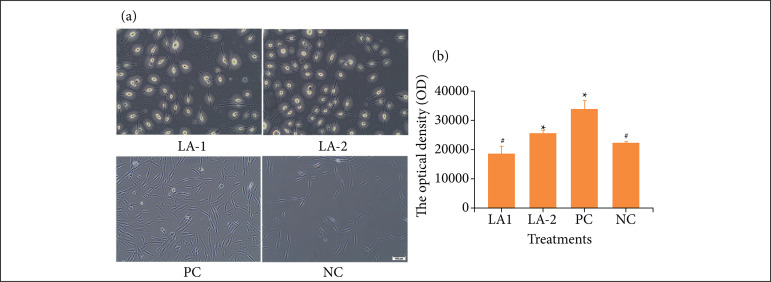
The effect of LA on HF proliferation. **(a)** The proliferation of HF under the microscope; **(b)** The effect of LA on the HF proliferation rate (the value of AB in the experimental group was subtracted from the LA blank group, n = 3).

LA-1: LA at a concentration of 180 mg/mL; LA-2: LA at a concentration of 112 mg/mL; PC: Positive control (serum-containing medium); NC: Negative control (SFM). Significance of the differences between the NC and HF were set at * p < 0.05 or ** p < 0.01. Significance of the differences between the PC and HF were set at ^#^ p < 0.05 or ^##^ p < 0.01.

When different concentrations of LA were applied to HF, the number of cells was different between each group (Fig. 3a). Microscope observation showed that in the positive control group, the spindle cells were tightly arranged. The cells in the experimental group with an LA concentration of 112 mg/mL was increased compared with the negative control group. In the experimental group with an LA concentration of 180 mg/mL, the number of cells was significantly lower than that in the negative control group (Fig. 3). The results of the AB test showed that an LA concentration of 112 mg/mL promoted cell proliferation, which was 15.07% higher than the negative control (n = 3, Fig. 3b).

### Effects of LA on FBW

#### Wound healing rate of porcine skin with FBW at different periods after treatment

**Figure 4:**
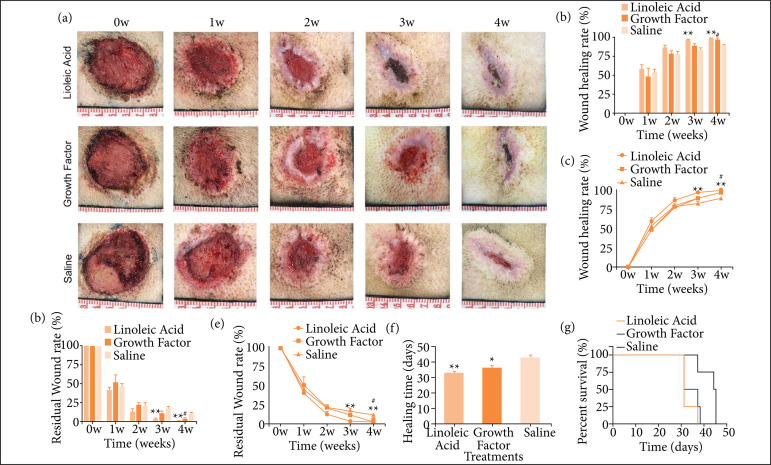
The effect of LA on the repair of burning wounds. **(a)** Wound repair from week 1 to week 4; **(b)** Wound healing rate from week 1 to week 4 (n = 4); **(c)** Change in the trend of the wound healing rate from week 1 to week 4 (n = 4); **(d)** Residual wound area rate from week 1 to week 4 (n = 4); **(e)** Change trend of residual wound area rate from week 1 to week 4 (n = 4); **(f)** Wound healing time (n = 4); and **(g)** Survival analysis of FBW of porcine skin after treatment.

The images of wounds were obtained at week 0, 1, 2, 3, and 4 after the administration, and the wound healing rate was quantitatively calculated using Image J (NIH, USA) (Fig. 4a and b). At week 1, 2, 3, and 4, the healing rate of burning wounds in the LA group increased gradually, which was 58.71% ± 5.06%, 85.98% ± 3.61%, 96.02% ± 2%, and 98.58% ± 0.78%, respectively; the healing rate of burning wounds in the EGF group increased gradually, which was 48.44% ± 11.28%, 78.16% ± 4.02%, 88.43% ± 2.65%, and 96.04% ± 2.25%, respectively. At week 1, 2, 3, and 4, the healing rate of burning wounds in the NS group increased gradually, which was 54.04% ± 4.47%, 78.42% ± 3.44%, 82.11% ± 3.37%, and 88.72% ± 1.73%, respectively. It can be seen from Fig. 4b and c, that the wound healing rate of the LA group was significantly higher at week 3 and 4 (n = 4, p < 0.05).

Significance of the differences between the groups of LA and NS were set at * p < 0.05 or ** p < 0.01. Significance of the differences between the groups of EGF and NS were set at ^#^ p < 0.05 or ^##^ p < 0.01.

#### Residual area rate of the FBW treated with LA

The residual wound area of the pig wound model was statistically analyzed after medication, and quantitatively calculated using Image J (NIH, USA) (Fig. 4a and d). At week 1, 2, 3, and 4, the residual wound area rate of burning wounds in the LA group decreased gradually, which was 41.29% ± 5.07%, 14.02% ± 3.61%, 3.98% ± 2%, and 1.42% ± 0.78%, respectively. The residual wound area rate of burning wounds in the EGF group decreased gradually, which was 51.57% ± 11.28%, 21.84% ± 4.01%, 11.57% ± 2.65%, and 3.96% ± 2.25%, respectively. At week 1, 2, 3, and 4, the residual wound area rate of burning wounds in the NS group decreased gradually, which was 45.96% ± 4.47%, 21.59% ± 3.44%, 17.89% ± 3.37%, and 11.28% ± 1.73%, respectively. It can be seen from Fig. 4d and e that the remaining wound area rate of the LA group was lower than that of the NS group from week 1 to week 4, and this difference was significant at week 3 and 4 (n = 4, p < 0.05).

#### Wound healing time of FBW treated with LA

The healing time after treatment with LA, EGF and NS were 32.5 ± 1.5 days, 36 ± 1.68 days, and 42.75 ± 1.93 days, respectively. It can be seen from Fig. 4f that the healing time of the LA and EGF groups were 10.25 days and 6.75 days, respectively, shorter than that of the NS group (n = 4, p < 0.05), whereas there was no significant difference in the wound healing time between the LA and EGF groups (n = 4, p > 0.05).

#### Survival analysis of FBW

Wound healing time was the time taken to reach complete epithelialization as shown in Table 2. The survival analysis curve of FBW of porcine skin after treatment was drawn using Prism (Dotmatics, USA) (Fig. 4g). The complete healing time of each wound was recorded. Among the 12 wound samples, the LA group healed 75% in 31 days and healed 100% in 37 days; the EGF group healed 50% in 31 days, 75% in 37 days, and healed 100% in 38 days; the NS group healed 25% in 37 days, 50% in 44 days, and 100% in 45 days (Fig. 4g).

**Table 2:** Survival process of healing time.

Sample	Treatment	Healing Days
1	LA	31
2	LA	37
3	LA	31
4	LA	31
5	EPG	31
6	EPG	37
7	EPG	38
8	EPG	31
9	Saline	44
10	Saline	37
11	Saline	45
12	Saline	45

LA: linoleic acid. EPG: epidermal growth factor.

Histological characteristics of FBW treated with LA

**Figure 5:**
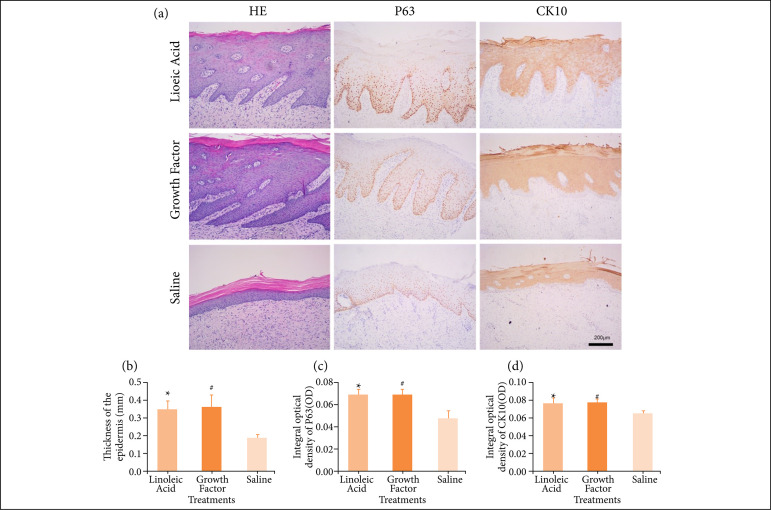
Effects of LA on protein expression in the burning wound model. **(a)** Comparison of epidermal thickness after wound healing and histological changes of the wound (10×); **(b)** Quantification of epidermal thickness (n = 5);**(c)** Changes in the expression of the P63 protein of the wound (n = 5); **(d)** Changes in theexpression of the CK10 protein of the wound (n = 5).

Hematoxylin-eosin (HE) staining was used to observe the epidermal thickness and this was quantified using Image J (NIH, USA). It can be seen from Fig. 5b that the epidermal thickness of the LA, EGF and NS groups was 0.35 ± 0.06 mm, 0.36 ± 0.07 mm, and 0.19 ± 0.02 mm, respectively. In the morphological comparison after healing with LA, the epidermal thickness of the LA and EGF groups was 0.16 ± 0.04 mm and 0.17 ± 0.05 mm, which are greater than that of the NS group, respectively (n = 5, p < 0.05).

#### Immunohistochemical analysis of FBW treated with LA

Immunohistochemical methods were used to investigate the proliferating epidermis and related proteins after wound repair (Fig. 5a). P63, a marker of cell proliferation, is mainly expressed in the nucleus of proliferating keratinocytes and is essential for maintaining the progenitor cell population necessary for epithelial development and morphogenesis^12^^,^ 13. CK10, a marker of cell differentiation, is expressed in the cytoplasm of differentiated keratinocytes^14^. The expression of P63 and CK10 proteins can show the proliferation and differentiation of keratinocytes during wound re-epithelialization and repair. Image-Pro Plus 6.0 software (Media Cybernetics, USA) was used to determine the integral OD of the P63 and CK10 proteins. It can be seen from the quantification that the OD of P63 in the LA, EGF, and NS groups was 0.07 ± 0.01, 0.07 ± 0.01, and 0.05 ± 0.01, respectively (Fig. 5c and d). Additionally, the OD of CK10 in the LA, EGF, and NS groups was 0.08 ± 0.01, 0.08 ± 0.00, and 0.07 ± 0.00, respectively. This showed that the expression of the P63 and CK10 proteins was stronger in the LA group than that in the NS group (n = 5, p < 0.05).

Significance of the differences between LA and NS was set at *p < 0.05. Significance of the differences between EGF and NS was set at ^#^ p < 0.05.

## Discussion

After a burn, human tissues will be damaged to a certain depth and area, forming nonhealing wounds due to various reasons, including infection and a weak immune system^2^. It also the focus and difficulty of treating burning patients^15^. Previous research showed that WO can significantly shorten the healing time of burning wounds, with low cost and no side effects^2^.

This study has further found that one of the main components in WO is bioactive fatty acid-LA, and the internal standard method showed that the LA content in WO is approximately 24% (Fig. 2). Consistent with other studies, reported that walnuts have a high content of fatty acids, the most important of which is LA^16^^–^ 18.

The effect of the major ingredient of LA was tested in vitro and in vivo. In the present study, the experimental results demonstrated that LA at a concentration of 112 mg/mL can promote HK and HF proliferation, which were 50.09% and 15.07% respectively higher than the negative control (Fig. 2b and Fig. 3b). In vitro cell experiments have showed that LA promotes wound healing by stimulating HK and HF proliferation, which is the main stage of wound healing^19^. Keratinocytes are the main cellular components of the epidermis and are responsible for its repair following damage through the process of epithelialization, which is a key indicator of healing^20^^,^ 21. HF are the key cells that support wound healing and play a key role in sound contraction and closure^22^^,^ 23. Both types of cells have an important impact on the final state of healing^23^. Herein, in vitro experiments showed that LA accelerates the repair of burning wounds by promoting keratinocyte and fibroblast proliferation, thus elucidating the mechanism of WO in promoting the healing of FBW.

A pig burning wound model was used to further verify the results of the above cell experiments in a functional manner. The pig skin structure is very similar to that of humans; thus, it has been widely used to establish animal models to understand the human skin^24^. EGF, NS, or LA were applied externally to the burning wounds of pigs, and the wounds were observed after week 1, 2, 3, and 4 to determine the progress of wound repair (Fig. 4). Compared with the negative control group, LA reduced the wound area and increased the wound healing rate (p < 0.05). The wound healing rate of LA was higher than that of the EGF group, but not statistically significant (p > 0.05). In addition, through the analysis of the healing time, LA can accelerate the wound healing time, which is 10.25 ± 0.43 days shorter than that of the NS group (Fig. 4f). Finally, wound survival statistics was used to describe the wound survival process. The degree of the healing in LA group reached 75% at 31 days, but only 25% at 37 days in NS group (Fig. 4g). Overall observation, LA showed fast healing speed and high healing rate in the process of promoting wound healing. Studies have reported that plant fatty acids rich in LA can improve wound healing and oral administration of LA can accelerated wound closure, which is consistent with our research^25^^–^ 27.

The results of the immunohistochemical analysis showed that LA can increase the protein expression in keratinocytes of P63 and CK10. Studies have shown that P63 can maintain the proliferation and renewal abilities of keratinocytes, and cells display an improved wound healing ability by upregulating P63 expression^28^^,^ 29. CK10 is a marker for the ultimate differentiation of HK^30^. The keratin layer can resist external abrasion and bacterial invasion, and plays a vital role in the quality of wound healing^31^. Immunohistochemical analysis showed that P63 and CK10 expression in the LA group was significantly increased, which promoted keratinocyte proliferation and differentiation, and therefore improved wound healing.

## Conclusion

This study investigated the effective components of WO, and revealed the mechanism of WO repair of FBW through both cellular and animal experiments. We found that LA, as one of the main components of WO, can effectively promote the healing of wound healing by stimulating cell proliferation and differentiation.
